# Bone and Extracellular Signal-Related Kinase 5 (ERK5)

**DOI:** 10.3390/biom14050556

**Published:** 2024-05-04

**Authors:** Lei Wen, Zirui Liu, Libo Zhou, Zhongcheng Liu, Qingda Li, Bin Geng, Yayi Xia

**Affiliations:** 1 Department of Orthopedics, The Second Hospital & Clinical Medical School, Lanzhou University, Lanzhou 730030, China; wenl2023@lzu.edu.cn (L.W.); 1202309011571@lzu.edu.cn (Z.L.); 120230901631@lzu.edu.cn (L.Z.); 120230901561@lzu.edu.cn (Z.L.); 120230901541@lzu.edu.cn (Q.L.); ery_gengb@lzu.edu.cn (B.G.); 2 Orthopedic Clinical Medical Research Center and Intelligent Orthopedic Industry Technology Center of Gansu Province, Lanzhou 730030, China; 3 Department of Orthopedics and Trauma Surgery, Affiliated Hospital of Yunnan University, Kunming 650032, China

**Keywords:** bone, homeostasis, ERK5, cancer

## Abstract

Bones are vital for anchoring muscles, tendons, and ligaments, serving as a fundamental element of the human skeletal structure. However, our understanding of bone development mechanisms and the maintenance of bone homeostasis is still limited. Extracellular signal-related kinase 5 (ERK5), a recently identified member of the mitogen-activated protein kinase (MAPK) family, plays a critical role in the pathogenesis and progression of various diseases, especially neoplasms. Recent studies have highlighted ERK5’s significant role in both bone development and bone-associated pathologies. This review offers a detailed examination of the latest research on ERK5 in different tissues and diseases, with a particular focus on its implications for bone health. It also examines therapeutic strategies and future research avenues targeting ERK5.

## 1. Introduction

Bones are crucial attachment sites for muscles, tendons, and ligaments, forming a vital part of the human body’s structural framework [1]. The health of mammalian bones is preserved through the dynamic interplay between bone formation, primarily driven by osteoblasts, and bone resorption, predominantly facilitated by osteoclasts [2]. This process involves a complex network of cells and regulatory pathways, with significant contributions from signaling molecules, such as hedgehog, notch, WNT, transforming growth factor beta (TGFβ), bone morphogenic protein (BMP), fibroblast growth factor (FGF), macrophage colony-stimulating factor (M-CSF), and receptor activator of nuclear factor-κB ligand (RANKL) [1,3]. Extracellular signal-related kinase 5 (ERK5), a recent addition to the mitogen-activated protein kinase (MAPK) family, plays a crucial role in various tissues and pathologies [4]. Although research on ERK5, particularly in the field of oncology, has been increasing, its role in bone biology has not been as thoroughly investigated. Bone biology includes processes, such as growth, development, and disease. This under-exploration is notable, especially when compared to the ERK1/2 signaling pathway. The ERK1/2 pathway is well studied and known to impact several cellular functions in skeletal cells. These functions include proliferation, differentiation, migration, survival, metabolism, and transcription [5]. Recent studies, however, indicate a growing interest in investigating ERK5’s role in bone-related conditions, notably osteoporosis and osteosarcoma. This review aggregates and synthesizes the existing literature on this topic.

## 2. Overview of Bone Development and Bone Homeostasis

### 2.1. Bone Formation

The maintenance and repair of mammalian bone homeostasis are contingent upon a delicate balance between bone formation, primarily orchestrated by osteoclasts, and bone resorption, predominantly governed by osteoclasts. This process involves a multitude of cells and intricate regulatory networks. The principal cells involved in osteogenesis are those of the bone lineage, including osteoblasts, osteocytes, and chondrocytes, whereas osteoclasts derive from the hematopoietic lineage [1,2]. In healthy adults, the synergistic action of osteoblast lineage cells and osteoclasts is essential for maintaining bone equilibrium. Disruption of this balance can lead to various bone disorders. Excessive bone formation relative to bone resorption can result in abnormal bone growth, such as heterotopic ossification. Conversely, osteoporosis arises when bone resorption supersedes bone formation. Additionally, exposure to carcinogens, genetic mutations, or aberrant gene expression can precipitate tumor development.

Osteoblasts originate from bone marrow mesenchymal cells, which are capable of differentiating into mature mesenchymal tissue cells, including those forming bone, cartilage, and fat, influenced by various factors [6]. The differentiation of osteoblasts encompasses three stages: osteoprogenitor cells, pre-osteoblasts, and osteoblasts. The transcription factor SRY-related high-mobility group box-containing9 (SOX9) plays a pivotal role in chondrocyte differentiation and chondrogenesis, initiating and driving chondrogenic differentiation through the activation of chondrogenic genes [1,7,8]. Recent research has identified Microtubule-Associated Serine/Threonine Kinase Family Member4 (Mast4) as crucial in determining the osteogenic or chondrogenic fate of mesenchymal stromal cells (MSCs). Mast4 induces the phosphorylation of SOX9 at serine 494, promoting its proteasomal degradation, while transforming growth factor-β1 (TGFβ1) counters this effect, enhancing SOX9 stability and favoring chondrogenesis. Conversely, enhanced stability of the Mast4 protein, through the Wnt-mediated inhibition of glycogen synthase kinase 3β (GSK-3β) and recruitment of Smad ubiquitination regulatory factor 1 (Smurf1), augments β-catenin nuclear localization and Runt-related transcription factor 2 (RUNX2) activity, fostering osteogenesis [9]. The expression of RUNX2 in cells signifies the emergence of pre-osteoblasts. Subsequently, WNT-β-catenin signaling influences these pre-osteoblasts, prompting the induction of osterix (OSX; also known as SP7), as the cells advance in their differentiation into osteoblasts. The expression of RUNX2 and OSX denotes the maturation of osteoblasts [3]. These osteoblasts can produce osteocalcin (OCN), alkaline phosphatase (ALP), and a substantial quantity of type I collagen (COL-I) to establish the bone’s framework. Hydroxyapatite minerals are deposited on this framework to solidify the bone structure [2,10]. Upon bone formation, some osteoblasts undergo apoptosis, while others secrete extracellular matrix components and integrate into the bone matrix, becoming osteocytes [11]. Bone formation occurs predominantly through two mechanisms: intramembranous ossification and endochondral ossification [12]. Intramembranous ossification is responsible for the formation of structures, such as the skull, mandible, maxilla, and clavicle. Conversely, endochondral ossification is the principal process in the growth of long bones, wherein mesenchymal stem cells evolve into chondrocytes. These chondrocytes progress through resting, proliferation, and hypertrophy stages. Hypertrophic chondrocytes facilitate the mineralization of the adjacent matrix, initiate vascular invasion by emitting vascular endothelial growth factor and other substances, and draw osteoclasts and bone marrow cells to the site. Subsequently, neighboring perichondral cells transform into osteoblasts. Following the apoptosis of hypertrophic chondrocytes, osteoblasts penetrate the cartilage matrix to generate new bone [2,13].

### 2.2. Bone Resorption

In vivo and in vitro experiments have established that osteoclasts originate from monocytes of the hematopoietic lineage [14], with their maturation governed by M-CSF and RANKL [15,16,17]. Osteoclast precursors are attracted to the bone by RANKL-expressing osteocytes, where they proliferate and evolve into mononuclear pre-osteoclasts before fusing to become multinucleated osteoclasts [14]. While osteoclasts may share a progenitor with macrophages and dendritic cells, only osteoclasts possess the capacity for bone resorption [15]. Characterized by their unique structure, osteoclasts feature multiple nuclei, a plethora of pleomorphic mitochondria, extensive vacuoles and lysosomes, and numerous stacked Golgi membranes [14]. Additionally, osteoclasts exhibit a complex, deeply invaginated, finger-like plasma membrane adjacent to the bone surface, termed ruffled borders. These cells can form a sealing zone beneath the ruffled border, secreting enzymes and acids into this space to irreversibly dissolve bone mineral [14].

## 3. ERK5 Overview

### 3.1. Discovery and Structure of ERK5

**Figure 1:**
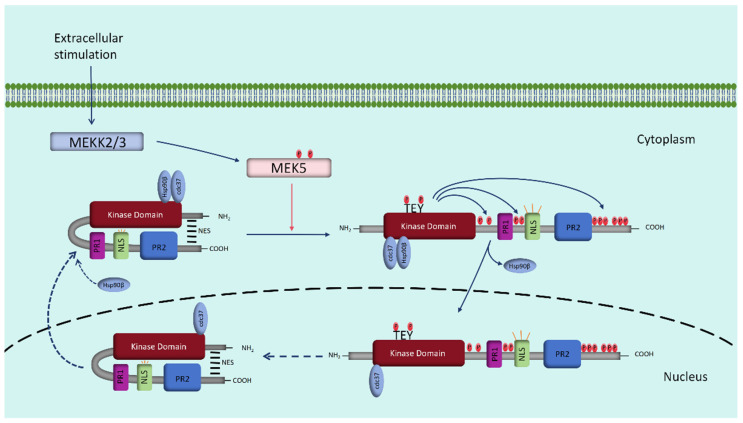
ERK5 localization and activation. In the resting state, ERK5 is present in the cytoplasm, and molecular interactions between the N-terminus and C-terminus of ERK5 place it in a folded state, which reduces the activity of the nuclear localization signal (NLS) or generates a nuclear export signal (NES) and binds to the cytoplasmic anchoring protein Hsp90. When the ERK5 TEY motif is phosphorylated and activated, the C-terminal half of ERK5 can autophosphorylate, inducing an unfolded state and dissociation from Hsp90, leading to NLS exposure and ERK5 nuclear translocation. When ERK5 is dephosphorylated in the nucleus, it resumes its folding transition and translocates to the cytoplasm. PR1: proline-rich 1; PR2: proline-rich 2.

ERK5, also known as mitogen-activated protein kinase 7 (MAPK7) or big MAP kinase 1 (BMK1), has a large carboxyl terminal. This kinase, a member of the mitogen-activated protein kinase (MAPK) family, was first identified in 1995 [18,19]. MAPKs, enzymes that exhibit high conservation across evolution, are found in organisms ranging from yeast to humans [20]. ERK5 functions via a three-tiered kinase cascade, similar to other MAPK family members [21]. At the apex of this cascade, the MAPK kinase kinase (MAPKKK) responds to extracellular signals, activating the MAPK kinase (MAPKK) through phosphorylation. The activated MAPKK then phosphorylates the threonine and tyrosine residues within the Thr-X-Tyr (TXY) motif of MAPK. Concurrently, the C-terminal tail of ERK5 undergoes autophosphorylation, altering its conformation and unfolding it. This change prompts the dissociation of heat shock protein 90 (Hsp90) from the ERK5-Cdc37 complex [22]. Subsequently, the activated ERK5 relocates to the nucleus, where it activates various downstream effector molecules, regulating transcription and other processes to elicit biological responses. Upon dephosphorylation, ERK5 reverts to its folded state and moves back to the cytoplasm [23] (Figure 1).

### 3.2. The Role of ERK5 in Different Tissues or Diseases 

ERK5 is extensively expressed in the heart, lungs, liver, and other organs, playing a crucial role in both physiological and pathological states. We summarize this as follows (Table 1):

#### 3.2.1. Heart and Vascular Endothelium

ERK5 mediates a range of biological processes, including cell survival, proliferation, differentiation, migration, apoptosis, and angiogenesis, across various tissues and organs [23,112]. It is crucial for cardiovascular development [35]. The complete deletion of the ERK5 gene in mice results in inadequate blood vessel and heart development, causing embryonic death between 9.5 and 10.5 days [32,33,34]. In healthy adult mice, ERK5 deletion leads to vascular endothelium disorders, resulting in extensive organ hemorrhage and mortality [34]. Intriguingly, while ERK5 deletion in the embryonic ectoderm, endothelial cells, or system causes embryonic death, mice with the ERK5 deletion in cardiomyocytes or neurons survive. Suppressing ERK5 in mouse vascular endothelial cells markedly reduces allograft tumor growth. Studies indicate a significant decrease in blood vessel density and a substantial reduction in the phosphorylation of ribosomal protein S6 (rpS6) at Ser235/236 [113]. Additionally, rpS6 is known to be crucial for both cell proliferation and the survival of vascular endothelial cells.

ERK5 has been established as a protector of the vascular endothelium [36]. NF-E2-related factor 2 (Nrf2), a crucial transcription factor, plays a significant role in the anti-atherosclerotic response under laminar flow. Under physiological conditions, vascular endothelial cells are subjected to the laminar stimulation of blood flow. The activation of ERK5 in these cells by laminar flow upregulates Nrf2, exerting anti-inflammatory and anti-apoptotic effects that safeguard endothelial cells against atherosclerosis and injury [37]. Concurrently, laminar flow-induced ERK5 activation prevents endothelial cell apoptosis by phosphorylating the BCL-2-associated death promoter (Bad) and inhibits the endothelial-to-mesenchymal transition, thereby preserving the vascular epithelium [38]. Zeste Homolog 2 (EZH2), the catalytic subunit of the Polycomb Repressive Complex 2, is pivotal in endothelial dysfunction. The antagonistic interaction between ERK5 and EZH2 is essential in regulating the endothelial-to-mesenchymal transition [39,114]. In research on post-hypertrophic cardiac remodeling, it was discovered that inhibiting ERK5 expression in neonatal rats diminishes myocyte enhancer factor 2 (MEF2) transcriptional activity and mitigates the hypertrophic response [115]. In a study by Cameron et al., constructing a mouse model of myocardial infarction revealed that ERK5 activation can stimulate platelets under ischemic conditions, facilitating infarct expansion and enhancing cardiac function post-myocardial infarction [26,27]. Further, it has been identified that post-myocardial infarction, left ventricular dysfunction exacerbates when ERK5 transcriptional activity is suppressed by diabetes-induced SUMOylation [28]. Additionally, apoptosis is observed. Liu et al. found a loss of ERK5 expression in the hearts of obese or diabetic animal models, leading to diminished cardiac contractility and mitochondrial dysfunction. However, restoring ERK5 expression ameliorated these abnormalities. Further investigations revealed that adequate ERK5 expression could prevent metabolic stress cardiomyopathy [30]. Bim functions as a critical pro-apoptotic protein; however, increased ERK5 expression suppresses Bim expression, thereby reducing cardiomyocyte apoptosis and highlighting ERK5’s protective role in the heart under cold stress [29]. Concurrently, another study showed that ERK5 inhibition promotes cardiomyocyte apoptosis [31]. Angiotensin II enhances ERK5 activation in the mouse myocardium, inducing cardiac hypertrophy [116]. Conversely, targeted ERK5 knockout reduces this hypertrophy. ERK5 activates MEF2, which, as demonstrated by Lee et al., promotes the expression of heparin-binding epidermal growth factor-like growth factor (HB-EGF)-induced cyclooxygenase-2 (COX-2), further inducing cardiac hypertrophy. This evidence underscores ERK5’s role in cardiac remodeling regulation [117].

#### 3.2.2. Nervous System

ERK5 is prevalent in nerve cells and plays a significant role in neurogenesis [118,119]. Numerous studies indicate ERK5’s neuroprotective properties [44,45,120]. Suzaki et al. showed that H(2)O(2) rapidly and significantly activates ERK5, enhancing MEF2C DNA-binding activity to protect brain cells from ischemic cell damage [40]. Glucagon-like peptide-1 (GLP-1) enhances ERK5 expression, mitigating oxidative harm to nerve cells and curbing neuronal apoptosis [41]. In the context of nervous system tumors, ERK5 facilitates glioma cell migration and invasion, whereas microRNA-429 (miR-429) inhibits these processes [46]. Van et al. reported that ERK5 is present in glaucomatous retinal ganglion cells and is involved in mediating retrograde neurotrophic signaling in the rat optic nerve [121]. The conditional activation of ERK5 enhances adult spatial learning and bolsters hippocampus-dependent long-term memory [42]. CX3C motif ligand 1 (CX3CL1) is released from the neuronal membrane following peripheral nerve injury and plays an important role in transmitting injury signals between neurons and microglia. CX3CL1 and CX3C chemokine receptor 1 (CX3CR1) facilitate nociceptive communication between neurons and microglia through ERK5-mediated microglial activation [43]. ERK5 activation in spinal microglia and dorsal root ganglion neurons is linked to the development of neuropathic pain, and inhibiting this pathway could alleviate pain following nerve injury [122]. Additionally, ERK5 is involved in regulating inflammatory diseases and neuropathic pain [123].

#### 3.2.3. Lung

Liang et al. demonstrated that ERK5 could counteract tobacco smoke (TS)-induced lung epithelial-to-mesenchymal transition (EMT), a process known to facilitate cancer occurrence, invasion, and metastasis [52]. Furthermore, an in vitro study indicated that sulforaphane (SFN) activates ERK5 to inhibit EMT in lung cancer cells [53]. ERK5 is a key protein in macrophage endocytosis. The glycoprotein ulinastatin (UTI) increases ERK5 levels, enhances endocytosis through the ERK5/Mer signaling pathway, and reduces lung inflammation and injury [47]. However, some studies present contrasting findings. Research utilizing A549 adenocarcinoma human alveolar basal epithelial cells showed that Biochanin A (the primary isoflavone component in chickpeas) might suppress PM2.5-induced ERK5 expression by targeting MEK5, showcasing anti-acute lung cell injury effects [124]. This discrepancy suggests that ERK5’s functions may vary across different cell types.

Some studies highlight the detrimental effects of ERK5. It facilitates TGFβ1-induced pulmonary fibrosis by increasing Smad3 acetylation [48]. The TGF-β1/Smad signaling pathway plays a key role in the pathogenesis of pulmonary fibrosis. The epidermal growth factor receptor (EGFR) can recruit mitogen-activated protein kinase kinase kinase 3 (MEKK3) in a tumor necrosis factor receptor (TNF-R)-associated factor 4 (TRAF4)-dependent manner, which activates ERK5 and supports the proliferation of non-small-cell lung cancer cells [50]. This activation leads to an increase in upstream stimulatory family 1 (USF1) transcription factor expression, which, in turn, upregulates focal adhesion kinase (FAK) expression, enhancing FAK signaling and promoting migration in non-small-cell lung cancer cells [54]. Additionally, ERK5 regulates lipid metabolism in small-cell lung cancer cells, increasing cell viability and tumor growth [51]. ERK5 signaling has been implicated in promoting migration and invasion in lung cancer and melanoma [54]. Treatment combining osimertinib with ERK5 or MEK5 inhibitors effectively curtailed the survival of osimertinib-resistant lung cancer cell lines, indicating ERK5’s role in drug resistance development in lung cancer cells [55]. Moreover, ERK5 can enhance the production of interleukin-6 (IL-6), aiding lung cancer cells in evading anti-tumor immune responses [57], and can also amplify the DNA damage response, increasing lung cancer cells’ resistance to radiotherapy [56]. Consequently, many researchers consider ERK5 a potential therapeutic target for lung tumors, with some studies validating the efficacy of targeting the MEK5/ERK5 pathway in lung cancer treatment [125].

#### 3.2.4. Breast

In breast cancer, ERK5 plays a crucial role in tumor formation and progression. Sawhney et al. established that ERK5 is instrumental in cell adhesion, movement, and metastasis, enhancing metastatic potential in breast and prostate cancer cells [70]. Mulloy et al. identified the cyclin D1 gene as a downstream target of the ERK5 cascade, highlighting ERK5’s involvement in cell cycle regulation [62]. The inhibition of ERK5 reduces cancer cell proliferation and increases sensitivity to anti-HER2 therapy [59,60]. Recent findings in breast cancer research demonstrate that ERK5 inhibition results in cell cycle arrest and amplifies the antiproliferative effects of anti-HER2 therapy in resistant cell lines [63]. Monlish et al.‘s research underscores ERK5’s role in promoting breast cancer cell proliferation and its interaction with the phosphoinositide 3-kinase (PI3K) pathway [61]. Numerous studies have shown that ERK5 contributes to tumor invasion and migration during breast cancer development. A kinome-wide high-content siRNA screen indicated that MEK5-ERK5 signaling is vital for EMT and metastasis in breast cancer cells [71]. Javaid et al. reported that ERK5 inhibition modulates EMT and reduces circulating tumor cells, thereby diminishing tumor invasiveness [64]. Similarly, Zhai et al. demonstrated that downregulating ERK5 suppresses EMT and curtails breast cancer tumor growth [65].

Transcriptional upregulation of MEK5 by signal transducer and activator of transcription 3 (STAT3) enhances the epithelial–mesenchymal transition in breast cancer cells, facilitating their invasion and metastasis [68]. ERK5 modulates the ECM and upholds its integrity, thus supporting triple-negative breast cancer (TNBC) cell growth and migration [69]. Xia et al. reported that ERK5 acts as a substrate for microRNA-143-3p (miR-143-3p), which downregulates ERK5 expression, thereby inhibiting the proliferation, migration, and invasion of breast cancer cells [58]. Furthermore, ERK5 fosters the invasion of TNBC cells by activating FAK, which is associated with tumorigenesis and migration [67]. ERK5 also plays a role in mediating drug resistance, with the combined inhibition of the PI3k/Akt and MEK5/ERK5 pathways reducing the viability of tamoxifen-resistant breast cancer cells [74]. However, some findings appear contradictory. Chen et al. demonstrated that ERK5 could inhibit epithelial–mesenchymal transition via the AKT/GSK3β signaling pathway [72]. Zuo et al. investigated Cdc42’s role in breast cancer, discovering that Cdc42 negatively regulates ERK5 phosphorylation, thus enhancing breast cancer cell migration and invasion [73]. They proposed that ERK5 expression might be inversely associated with the advancement of certain breast tumors.

#### 3.2.5. Kidney and Liver

ERK5 is essential for renal tubulogenesis [35], predominantly expressed in mesangial cells [126,127] and proximal tubular cells within the kidney [128]. Kato et al. established that EGF effectively activates ERK5, which is crucial for EGF-induced cell proliferation and cell cycle progression [129]. The downregulation of ERK5 curtails proliferation in human kidney-derived mesangial cells, EGF-stimulated cell contraction, and diminishes TGFβ1-induced collagen I expression [75]. Additionally, ERK5 facilitates platelet-derived growth factor (PDGF)-induced migration of glomerular mesangial cells [78]. Research using a mouse model of renal ischemia–reperfusion injury highlighted ERK5’s renal protective role, noting that kidney ERK5 possesses a smaller apparent molecular mass, indicating a variant form [77]. ERK5 also plays a role in various renal pathological conditions. Badshah et al. discovered that ERK5 inhibitors could thwart TGFβ1-induced podocyte proliferation without affecting apoptosis [76]. Elevated glucose levels trigger ERK5 activation in rat glomeruli and mesangial cells, suggesting ERK5’s involvement in diabetic nephropathy’s onset and progression [126]. Furthermore, ERK5 promotes chronic glomerular fibrosis in RAS-induced glomerulonephritis [82]. In the context of renal tumors, Kanno et al. observed that upregulated ERK5 promotes proliferation and suppresses apoptosis in renal cancer cells. The inhibition of ERK5 led to elevated expression of the cyclin-dependent kinase inhibitor p21 and reduced levels of the anti-apoptotic molecule B-cell lymphoma 2 (BCL2) [79]. Arias et al. confirmed that increased ERK5 levels foster proliferation, invasion, and metastasis in clear cell renal cell carcinoma, with a positive correlation between ERK5 expression and the disease’s invasiveness and metastasis in fresh human samples [81]. Another study identified a connection between ERK5 and enhanced metastasis and advanced disease stages [130]. A clinical investigation underscored ERK5’s prognostic significance in renal cell carcinoma, where patients with elevated ERK5 expression experienced poorer outcomes [131].

The ERK5 signaling pathway modulates the cell cycle through various mechanisms and influences liver cell proliferation [132]. Hepatic stellate cells, critical in liver injury repair, are regulated by ERK5, which enhances their proliferation and diminishes their migration capacity in response to PDGF [83]. In tissue samples from hepatocellular carcinoma patients, ERK5 expression in tumor and adjacent non-tumor tissues was elevated compared to normal liver tissue. Further research indicated that ERK5 accelerates cancer cell proliferation and cell cycle progression. The ERK5-specific inhibitor XMD8-92 has been shown to inhibit these activities [80].

#### 3.2.6. Skin

ERK5 plays a crucial role in cancer-related inflammation during epidermal carcinogenesis [133]. Additionally, TRAF1 expression increases ERK5 ubiquitination at K184, subsequently activating Activator Protein-1 (AP-1), which facilitates solar UV-induced skin cancer [134]. Numerous studies have highlighted its association with melanoma. Investigations into ERK5 in skin tumors reveal that melanoma exploits the inflammatory tumor microenvironment to its advantage [86,87,88], thereby enhancing tumor cell proliferation [87,89]. ERK5 activation in macrophages leads to the phosphorylation of signal transducer and activator of transcription 3 (STAT3), thus promoting melanoma growth. Conversely, inhibiting ERK5 can reprogram macrophages into an anti-tumor phenotype, effectively curbing melanoma and cancer graft growth [88]. Elevated ERK5 expression not only augments melanoma migration, invasion, and lung metastasis [54] but also supports the anti-aging properties of melanoma cells. Targeting ERK5 can trigger p21-mediated cellular senescence in melanoma [90]. Furthermore, Lee et al.’s research further clarifies ERK5’s role in mediating drug resistance [135]. While most existing studies indicate ERK5’s role in promoting melanoma development, it has been noted that phosphorylation of ERK5 at the S496 residue may facilitate an exercise-induced inhibition in tumor growth [86]. Additionally, Wu et al. reported that IL-17 (IL-17A) sustains a chronic inflammatory milieu, conducive to cutaneous squamous cell carcinoma formation, with IL-17 stimulating keratinocyte proliferation and tumor genesis via the TRAF4-ERK5 axis [87]. Tumor-associated macrophages (TAMs) significantly impact the tumor microenvironment (TME). Based on extensive research across various malignancies, Giurisato et al. determined that TAM proliferation is a common mechanism in cancer. Subsequent research revealed that ERK5 inhibits the differentiation of macrophages by suppressing p21 expression, thus preserving their proliferative capacity. Furthermore, it was found that ERK5-mediated macrophage proliferation enhances the in vivo metastasis of melanoma [136]. 

#### 3.2.7. Hematopoietic System

ERK5 plays a pivotal role in leukemia cell survival and influences the monocyte differentiation of human myeloid leukemia cells [91]. In vitro studies show that inhibiting the MEK5/ERK5 pathway markedly diminishes the proliferative and colony-forming capacities of primary chronic myelogenous leukemia (CML) cells [93]. Additionally, the ERK5 pathway impacts the terminal differentiation of myeloid leukemia cells triggered by 1α, 25-(OH)2 vitamin D3. ERK5 inhibitors can also induce G2 phase arrest in acute myeloid leukemia (AML) cells, thus curtailing cell proliferation [94]. Fms-like tyrosine kinase-3 (FLT3), with its most prevalent activating mutation being internal tandem duplication (ITD), is a growth factor receptor. MEK5/ERK5 pathways counteract apoptosis in FLT3-ITD leukemia cells, with increased apoptosis observed upon ERK5 inhibition [137]. Hartmann et al. identified ERK5 as a target of miR-143, which suppresses ERK5 expression, thereby hindering AML cell growth and promoting apoptosis [97]. Suzuki et al.’s findings further corroborate that ERK5 bolsters leukemia cells’ resistance to apoptosis triggered by extrinsic pathways [96]. ERK5 also mediates CML cell resistance to imatinib, with Erk5 deletion significantly impeding the cell cycle progression in imatinib-treated cells [97]. Consequently, many researchers advocate targeting the ERK5 pathway as a novel strategy to address human chronic myelogenous leukemia stem cells [138].

#### 3.2.8. Prostate

The activation of ERK5 stimulates the entry of prostate cancer cells into the S phase of the cell cycle, thereby enhancing cell proliferation [98]. Mehta et al. confirmed the involvement of the MEK5/ERK5 pathway in the regulation of prostate cancer cell proliferation [139], potentially linked to the initiation of MEK5/ERK5 signaling and activation of DNA replication licensing pathways in the prostate [140]. Additionally, research has shown that phthalates activate ERK5, further promoting prostate cancer cell proliferation [100]. Clapé et al. noted that ERK5 is intimately associated with tumor cell proliferation and migration, and that miR-143 can reduce ERK5 expression, leading to cell proliferation arrest and tumor growth inhibition in mice [99]. McCracken et al. observed a significant increase in ERK5 levels in prostate cancer compared to benign prostatic hyperplasia, with subsequent findings indicating that ERK5 elevates tumor cell proliferation, invasion, and migration [101]. Ramsay et al. found that ERK5 boosts the development of invasive pseudopodia and in vivo metastasis in prostate cancer cells [102]. Moreover, Loveridge et al. suggested that the response of prostate cancer to immune checkpoints might be influenced by ERK5’s role in promoting T-cell infiltration in prostate cancer [141].

#### 3.2.9. Other Tissues

A limited number of studies have elucidated the role of ERK5 in various tissues and diseases. ERK5 is known to regulate the proliferation of esophageal cancer cells, and its knockdown can mitigate this effect, a finding derived from analyzing samples from patients with squamous cell lung cancer and esophageal cancer [49]. Zhou et al. reported that enhancing the ERK5 pathway facilitates the viability and metastasis of esophageal squamous cell carcinoma cells [103]. Sticht et al. performed gene expression profiling on healthy oral mucosa and oral squamous cell carcinoma tissues, discovering a strong association between high ERK5 expression, advanced cancer stage, and lymph node metastasis [105]. Oxytocin receptor high-expressing (OXTRHigh) stromal fibroblasts regulate oral squamous cell carcinoma invasion via ERK5 signaling [104]. Gentilini et al. demonstrated that ERK5 promotes proliferation, migration, and invasion in cholangiocarcinoma; silencing ERK5 reduces the expression of angiogenic factors VEGF and angiopoietin 1, subsequently inhibiting these phenotypes [106]. ERK5 has been implicated in the regulation of small intestinal tumor development [142]. Research in colorectal cancer indicated that the loss of ERK1/2 in intestinal epithelial cells led to nutrient absorption impairments, disrupted epithelial cell migration, and secretory cell differentiation anomalies, albeit without hindering the proliferation of intestinal epithelial cells. Subsequent investigations revealed that ERK5 experiences enhanced activation, compensating for the proliferation of intestinal epithelial cells [108]. Lv et al. identified a significant function of ERK5 in zebrafish, revealing its crucial role in intestinal development and integrity maintenance. In zebrafish larvae, the knockout of erk5 results in underdeveloped intestinal walls and increased permeability [109]. Additionally, ERK5 exacerbates tobacco smoke-induced gastric epithelial-to-mesenchymal transition in mice, a process reversible through ERK5 inhibition [143]. The overexpression of Special AT-rich sequence-binding protein 2 (SATB2) diminishes ERK5 expression, thereby curtailing gastric cancer proliferation and migration [110]. Although sparingly discussed [144,145], ERK5 is suggested to facilitate tumor growth in ovarian cancer by upregulating type II collagen expression and enhancing cancer cell proliferation, invasion, and migration [111]. ERK5 plays a role in the pathogenesis and progression of various cancers [84,146]. Charlson et al. posited that ERK5 could act as an auxiliary mediator, conveying stimulatory signals from diverse tumor promoters and participating in the regulation of tumor development [147]. Numerous studies indicate that the ERK5 signaling pathway contributes to epithelial–mesenchymal transition, invasion, and metastasis across different cancers through varied mechanisms, with microRNA and knockdown strategies capable of inhibiting these processes [148]. Erazo et al. discovered that Cdc37 overexpression promotes Hsp90’s dissociation from the ERK5-Cdc37 complex and facilitates the nuclear translocation of non-phosphorylated yet transcriptionally active ERK5, suggesting that ERK5 may promote tumor progression without specific activating enzymes in some cancer types [22].

In addition, there are relatively few studies that highlight the distinct role of ERK5. Whole-exome sequencing results suggest that ERK5 could be linked to susceptibility to autism spectrum disorder [149]. Research indicates that ERK5 exerts a pro-inflammatory effect in primary human endothelial cells and monocytes, with its inhibition reducing inflammation and enhancing survival in endotoxemic mice [150]. In 2017, Yang et al. reported that ERK5 could facilitate thrombosis [151]. An in-depth analysis of homeobox genes in Hodgkin lymphoma cell lines suggests the potential involvement of ERK5 in the development of Hodgkin lymphoma [152]. Devost et al. unveiled a novel function of ERK5, demonstrating its role in inhibiting oxytocin-induced COX-2, thereby preventing uterine muscle contractions, an activity dependent on ERK5 [153]. Additionally, ERK5 is implicated in the regulation of decidualization, proliferation, and migration of human endometrial stromal cells, mediated through a lysine-deficient protein kinase 1 (WNK1)-dependent pathway [154].

## 4. The Role of ERK5 in Bones

### 4.1. ERK5 and Bone Metabolism (Figure 2)

#### 4.1.1. Bone Formation

In adult bone marrow mesenchymal stem cells (BM-MSCs), ERK5 plays a crucial role in regulating bone homeostasis, maintaining a dynamic balance by preventing excessive osteogenesis through the Erk5-Smurf2Thr249 axis [155]. Li et al. were pioneers in demonstrating that fluid shear stress (FSS) enhances ERK5 phosphorylation in MC3T3-E1 cells, elevates cyclin D1 expression, and stimulates osteoblast proliferation [156]. The integrity of the cytoskeleton is essential for FSS-induced ERK5 activation, as its disruption significantly reduces ERK5 phosphorylation [157]. Intermittent FSS stress activation of ERK5 increases ALP activity and boosts the protein expression of OPN and OCN, thereby fostering osteoblast differentiation. Additionally, ERK5 influences the expression of RUNX2 triggered by FSS [158]. Subsequent studies revealed that FSS could mitigate TNF-α-induced apoptosis in MC3T3-E1 cells, with activated ERK5 promoting AKT phosphorylation, thereby activating FoxO3 to deter apoptosis [159]. Contrasting reports exist where the inhibitor BIX02189 of MEK5 significantly decreases ERK5 phosphorylation, inhibiting osteoblast proliferation but promoting differentiation in MC3T3-E1 cells. Both RNA interference knockdown of ERK5 and adenoviral overexpression have corroborated these findings [160]. The exact cause of these discrepancies, whether due to the absence of FSS loading or other unknown mechanisms, requires further investigation. Zhang et al. conducted extensive research to decipher the influence of the G protein αq subunit (Gαq) on osteoblast proliferation, demonstrating that FSS induces osteoblast proliferation via the Gαq-ERK5 pathway [161]. FSS also stimulates osteoblast proliferation by enhancing Nuclear Factor of Activated T-cells 1 (NFATc1), augmenting ERK5 phosphorylation and elevating the expression of E2F transcription factor 2 (E2F2) and cyclin E1 [162]. Furthermore, Kruppel-like factor 4 (KLF4) can inhibit the proliferation of osteoblast lineage cells, but FSS upregulates ERK5, reducing KLF4 expression and promoting osteoblast lineage cell proliferation [163]. Wang highlighted an additional mechanism, where an FSS-induced reduction in miR-140-5p expression activates the VEGFA/ERK5 signaling pathway, thus enhancing osteoblast proliferation [164].

A study on human bone marrow-derived multipotent progenitor cells (MPCs) revealed that ERK5 negatively regulates their chondrogenesis. RNA interference knockdown of MEK5 and ERK5 in MPCs leads to the increased expression of SOX9 and COL2A1, contrasting with the regulation seen in the ERK1/2 kinase cascade [165]. In vitro experiments demonstrated that a shear force of 2dyn/cm^2^ induces peroxisome proliferator-activated receptor gamma transcriptional activity via ERK5 [166], increasing Kruppel-like factor 4 (KLF4) transcription. KLF4, a versatile transcription factor [167], subsequently reduces IL-1β-stimulated nuclear factor-κB activation, thereby offering protection to cartilage [7]. SOX9, a high-mobility-group (HMG) domain transcription factor, is recognized as a crucial regulator of chondrocyte differentiation and chondrogenesis [7]. In ERK5-deficient mesenchymal cells, SOX9 expression notably increases, as do the protein levels of Smad1, Smad2, and Smad3, with SOX9 induction. ERK5 phosphorylates Thr at the Smurf2 site Thr to activate Smad ubiquitination, which regulates the Smurf1-dependent proteasomal degradation of Smad1, hence impeding chondrogenic differentiation [168]. Further research indicated that ERK5 downregulates Col2A1 and SOX9 expression, a process inhibited by an ERK5 inhibitor [169]. Wu et al. performed an ERK5 knockout study in mouse chondrocytes, resulting in kyphosis and osteopenia. They discovered that ERK5 deficiency leads to increased MEF2C expression, activating PI3K/AKT signaling, inducing chondrocyte hypertrophy, and impairing vertebral body ossification [170]. ERK5 is crucial for chondrocyte proliferation, survival, and differentiation during growth plate development [171]. Mice with chondrocyte-specific ERK5 knockout exhibit postnatal limb shortening, with studies showing reduced chondrocyte proliferation and increased apoptosis in the central proliferative layer (the most hypoxic area) and downregulated hypertrophic differentiation markers below the abnormal cell apoptosis layer. The underlying mechanism may involve ERK5’s inability to modulate hypoxia-inducible factor-1α (HIF1α) signaling, crucial for adapting to hypoxic conditions [171]. In human mesenchymal stem cells, ERK5 activation upregulates parathyroid hormone-like hormone (PTHLH) expression, a key regulator of calcium and bone homeostasis [172]. PTHLH is known to enhance bone matrix strength by influencing bone mineralization without affecting osteoclast generation [173]. During osteoblast differentiation, PTHLH expression declines, alongside a reduction in ERK5 phosphorylation [172]. 

#### 4.1.2. Bone Resorption

Studies on ERK5 in osteoclasts are few, and the results are inconsistent. Osteoclasts, originating from bone marrow macrophages, are pivotal in maintaining bone equilibrium by resorbing mineral and organic bone components [174]. ERK5 plays a critical role in osteoclast differentiation; Amano et al. discovered that M-CSF activates ERK5, subsequently inducing the transcription factor c-Fos and steering RAW264.7D clone cells toward osteoclast differentiation—a process inhibited by MEK5 or ERK5 blockers [175]. Intriguingly, a study by Loveridge et al., which specifically knocked out ERK5 in mouse prostates, found that all ERK5-deficient mice exhibited pronounced thoracic spine curvature deformation and reduced trabecular bone volume. They revealed that ERK5 knockout increases osteoclast numbers and the expression of osteoclast marker genes [176]. There is also an alternative view that FSS can diminish the expression of nuclear factor of activated T-cells 1 (NFATc1) and its downstream effectors, matrix metalloproteinase-9 (MMP-9), cathepsin K (CTSK), and tartrate-resistant acid phosphatase (TRAP), inhibiting osteoclast differentiation by elevating ERK5 [177]. Chemerin, an adipokine with elevated levels in obesity, type 2 diabetes, and osteoporosis patients, was found to heighten ERK5 expression, enhancing actin ring formation and bone resorption activity in mature osteoclasts, without impacting osteoclast differentiation and formation [178].

#### 4.1.3. Osteoporosis

A study incorporating genetic sequencing and in vitro experiments in patient cohorts revealed that rare coding variants of ERK5 are susceptibility genes for Adolescent Idiopathic Scoliosis (AIS). The researchers found that three ERK5 mutants interfered with ERK5 nuclear translocation, a discovery validated using zebrafish models [179]. ERK5 knockout in zebrafish led to the manifestation of scoliosis, underscoring the necessity of ERK5 in skeletal development and functional maintenance. Osteoporosis arises when bone anabolic activity is surpassed by catabolic activity, often linked to diminished osteoblast production and functionality [2]. Mechanical stimuli play a pivotal role in bone homeostasis [23]. Guevara et al. utilized finite element analysis to ascertain that stress distribution during bone development influences local epiphyseal structure growth and histological alignment with the growth plate, illustrating the correlation between mechanical stress and bone mass [180]. Conditions like fracture fixation and prolonged bed rest lead to osteoporosis due to the absence of stress stimulation [181]. Similar to vascular endothelial cells, shear stress in interstitial fluid and blood vessels is integral to maintaining bone tissue equilibrium and facilitating bone repair [36]. Previous studies have established that fluid shear force enhances ERK5 expression, which, in turn, modulates osteoblast and osteoclast activity, playing a role in osteoporosis development. Astronauts experience bone loss under microgravity, posing a significant challenge in space exploration [182]. Research indicated that simulated microgravity activates the ERK5/NF-κB/IL-8 axis, leading to the proliferation and increased migration of pancreatic cancer cells [183]. The influence of ERK5 on osteoporosis in microgravity conditions is a subject of ongoing research in our laboratory. Additionally, the expression levels of ERK5 in normal versus degenerated nucleus pulposus tissue vary significantly, suggesting ERK5’s involvement in the regulation of human nucleus pulposus tissue degeneration, although the precise mechanism remains to be elucidated. 

### 4.2. ERK5 and Bone Neoplasms

Osteosarcoma is a prevalent malignant tumor among children and adolescents [184,185]. ERK5 has been found to enhance the growth, proliferation, and migration of osteosarcoma cells, without diminishing cell viability. Kim et al. utilized siRNA to suppress ERK5 in osteosarcoma cell lines, assessing its biological role. Their findings indicated that ERK5 expression stimulates MMP-9 expression and regulates the invasion of osteosarcoma cells; however, silencing ERK5 does not impact cell proliferation [186]. Yue further demonstrated that knocking down ERK5 in osteosarcoma cell lines could enhance Slug signaling and MMP-9 expression, thereby reducing tumor cell migration and invasion [187]. Single-cell sequencing on primary bone cancer revealed that the ERK5/MMP9 pathway promotes cancer cell proliferation, colony formation, migration, tumor growth, and lung metastasis, with the RNA interference silencing of ERK5 mitigating these effects [188]. Research analyzing osteosarcoma samples before and after chemotherapy suggested that monotherapy targeting osteosarcoma is insufficient, proposing that ERK5-specific inhibitors could complement multimodal treatments [189]. Tesser et al. supported this approach, discovering that combining major chemotherapy agents (cisplatin, doxorubicin, and methotrexate) with ERK5 silencing significantly boosts the inhibitory impact on ERK5, presenting a potential therapeutic strategy [190]. Moreover, ERK5 serves as a valuable marker for assessing treatment and prognosis in osteosarcoma [191]. Lopes et al. observed overexpression of the ERK5 gene in pre-chemotherapy samples compared to post-treatment and normal bone specimens, associating high ERK5 expression with adverse therapeutic outcomes and lower survival rates in osteosarcoma patients [192]. Numerous studies corroborate the therapeutic potential of targeting ERK5 in various malignancies [193,194]. Tusa’s team demonstrated that inhibiting the MEK5/ERK5 pathway reduces CML cell growth [93]. Bioinformatics and luciferase reporter assays confirmed ERK5’s analogous role in osteosarcoma, with microRNA-143 targeting and inhibiting ERK5 to decrease osteosarcoma cell proliferation and migration [195]. In vitro studies verified that ERK5 promotes proliferation, migration, and invasion in human osteosarcoma cell lines, whereas microRNA-187 suppresses these processes by targeting ERK5, with ERK5 overexpression countering this effect. ERK5 has been identified as a key regulator of macrophage polarity; inhibiting ERK5 curtails STAT3-induced gene expression, thus reprogramming macrophages into an anti-tumor state [88]. Interaction between osteosarcoma cells and the surrounding TME is crucial for tumor growth and metastasis. TAMs play a vital role within the tumor matrix, significantly influencing the surrounding microenvironment and participating in the regulation of osteosarcoma progression and bone balance [196]. This suggests that ERK5 may perform similar roles in the osteosarcoma microenvironment and tumor-associated macrophages (TAMs), making it a compelling subject for further research.

In myeloma, IL-6 can activate Erk5 independently of Ras and tyrosine–protein kinase Src(Src), thereby enhancing myeloma cell proliferation [197]. TG02 (Zotiraciclib), a novel pyrimidine-based multi-kinase inhibitor, can inhibit ERK5 activity, leading to cell cycle arrest in multiple myeloma cells, thereby impeding proliferation and promoting apoptosis [198].

### 4.3. Drugs

Osteoporosis is characterized by bone loss and the deterioration of bone tissue structure, increasing susceptibility to fractures in patients [199]. Current osteoporosis treatments primarily comprise bone resorption inhibitors (such as calcium, estrogen, calcitonin, bisphosphonates, and vitamin D) and bone formation stimulants (such as fluoride, parathyroid hormone, vitamin K2, and growth factors). Research on anti-osteoporosis drugs also encompasses numerous synthetic compounds [200,201], with only a limited number of studies reporting on drugs that influence ERK5 (Table 2). Adam et al. discovered that bisphosphonates, in addition to inducing osteoclast apoptosis and offering anti-bone resorption benefits, can activate ERK5 in primary human endothelial cells and osteoblasts, promoting osteogenic differentiation and mineralization [172]. He et al. found that mangiferin, a xanthone glucoside extracted from plants, enhances the osteogenic differentiation of MC3T3-E1 cells via the receptor tyrosine kinase AXL/ERK5 axis and alleviates osteoporosis in ovariectomized (OVX) mice [202]. Vildagliptin, commonly used to manage type 2 diabetes, was recently highlighted by He et al. for its novel skeletal system mechanism. It stimulates the formation and differentiation of precursor osteoblasts and BMSCs through the GAS6/AXL/ERK5 axis while concurrently inhibiting osteoclast differentiation [203]. TG02 inhibits cyclin-dependent kinases (CDKs) 1, 2, and 9, as well as ERK5 activity, thereby suppressing myeloma cell proliferation [198].

Given that most research underscores ERK5’s role in fostering cancer progression, current drug development targeting ERK5 predominantly focuses on inhibitors. Presently, XMD8-92 [204,205], BIX02188, and BIX02189 [206] are extensively utilized, alongside various natural or synthetic compounds, largely informed by findings from other cancer studies [148,198,207,208,209,210,211,212,213,214,215,216]. Although significant advancements have been made in targeted osteosarcoma therapy in recent years [217], research on targeting ERK5 in osteosarcoma treatment remains scarce.

## 5. Summary and Outlook

Currently, the roles of ERK5 in cancer and targeted therapy remain the primary directions for future research. Investigations into ERK5’s function in bone are nascent, with numerous unresolved questions. For instance, the specific mechanisms through which ERK5 regulates the differentiation, proliferation, and apoptosis of bone cells remain to be elucidated. Additionally, the underlying processes by which ERK5 inhibits osteoclast differentiation, while simultaneously being essential for this process, require further clarification. Given ERK5’s dual role in both safeguarding bone and facilitating the progression of osteosarcoma and other cancers, strategizing its use as a therapeutic target for bone diseases continues to be a topic of significant merit.

## Figures and Tables

**Figure 2:**
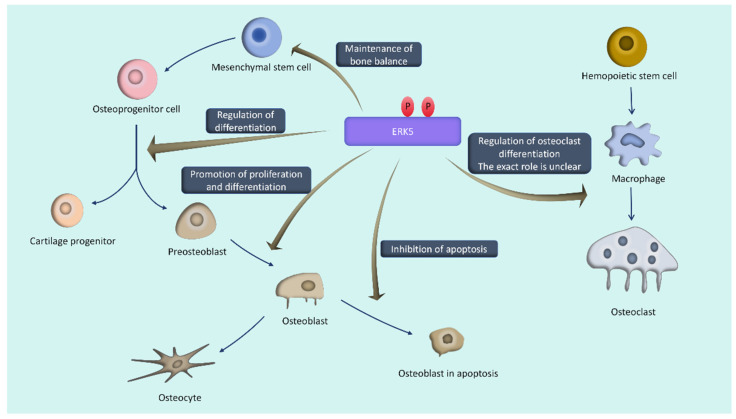
The role of ERK5 in maintaining bone homeostasis.

**Table 1:** The role of ERK5 in different tissues or diseases.

Distribution	Function	Disease	References
Heart	Maintain endothelial integrity Decrease cardiac endothelial cell permeability	-	[24]
	Protect myocardium Improve heart function	Myocardial infarction	[25,26,27]
		Myocardial infarction combined with diabetes	[28]
		Myocardial damage caused by hypothermia	[29]
		Metabolic stress-induced cardiomyopathy	[30]
	Inhibit cardiomyocyte apoptosis	Myocardial infarction	[31]
Vascular endothelium	Developmentally necessary	-	[32,33,34,35]
	Protect endothelial cells	-	[36]
	Anti-inflammation, inhibition of apoptosis	Atherosclerosis	[36,37,38]
	Inhibit endothelial-to-mesenchymal transition	Atherosclerosis	[37,39]
Nervous system	Protect brain cells	Ischemia–reperfusion injury	[40]
	Inhibit oxidative damage Inhibiting neuronal apoptosis	Traumatic brain injury	[41]
	Promote learning ability and memory	-	[42]
	Mediate damage signaling	Neuropathic pain	[43]
	Protect neurons from cell death	Oxidative stress	[44,45]
	Promote tumor cell migration	Invasion glioma	[46]
Lung	Promote resolution of inflammation	Acute lung injury	[47]
	promote pulmonary fibrosis	Pulmonary fibrosis	[48]
	Promote proliferation	Squamous cell lung cancer	[49]
	Promote cell proliferation	Non-small cell lung cancer	[50]
	Increase cell viability	Lung cancer	[51]
	Inhibit epithelial–mesenchymal transition	Lung cancer	[52,53]
	Promote invasion and migration	Lung cancer	[54]
	Promote drug resistance	Lung cancer	[55]
	Increase radiotherapy resistance	Lung cancer	[56]
	Promote immune evasion	Lung cancer	[57]
Breast	Promote proliferation	Breast cancer	[58,59,60,61,62,63]
	Promote invasion and migration	Breast cancer	[63,64,65,66,67,68,69,70,71]
	Inhibit epithelial–mesenchymal transition	Breast cancer	[72]
	Inhibits migration and invasion	Breast cancer	[73]
	Promote drug resistance	Breast cancer	[74]
Kidney	Necessary for renal tubule development	-	[35]
	Promote proliferation and contraction of renal mesangial cells	Kidney disease	[75]
	Promote podocyte proliferation	Diabetic nephropathy	[76]
	Protect the kidneys	Renal ischemia–reperfusion injury	[77]
	Promote mesangial cell migration	Chronic kidney disease	[78]
	Promote cancer cell proliferation	Kidney cancer	[79,80,81]
	Promote glomerular fibrosis	Glomerulonephritis	[82]
	Promote invasion and migration	Kidney cancer	[80]
		Clear cell renal cell carcinoma	[81]
	Inhibit cancer cell apoptosis	Kidney cancer	[79]
Liver	Promote hepatic stellate cell proliferation and inhibit migration.	Liver damage	[83]
	Promote cancer cell migration	Liver cancer	[84]
	Promote cancer cell proliferation Promote cell cycle progression	Hepatocellular carcinoma	[80,85]
Skin	Promotes an inflammatory tumor microenvironment	Melanoma	[86,87,88]
	Promote proliferation of melanoma	Melanoma	[87,89]
		Cutaneous squamous cell carcinoma	[87]
	Promote migration, invasion and lung metastasis	Melanoma	[54]
	Anti-cell aging	Melanoma	[90]
Hematopoietic system	Helps leukemic cells survive	Leukemia	[91]
	Regulates monocyte differentiation of human myeloid leukemia cells	Myeloid leukemia	[92]
	Promote proliferation	CML	[93,94]
		AML	[95]
	Inhibit apoptosis	AML	[95,96]
	Promote drug resistance	CML	[97]
Prostate	promote proliferation	Prostate cancer	[98,99,100,101]
	Promote migration and invasion	Prostate cancer	[101,102]
Esophagus	Promote proliferation	Esophageal cancer	[49]
	Prognosis related	Esophageal cancer	[103]
Oral cavity	Promote cancer invasion	Oral squamous cell carcinoma	[104]
	Associated with advanced cancer stage and metastasis	Oral squamous cell carcinoma	[105]
Bile duct	Promote proliferation, migration and invasion	Cholangiocarcinoma	[106,107]
Intestine	Promote proliferation	Rectal cancer	[108]
	Promote intestinal development and maintain intestinal integrity	Intestinal agenesis (zebrafish)	[109]
Stomach	Promote proliferation and migration	Gastric cancer	[110]
Ovary	Promote cancer proliferation, invasion and migration	Ovarian cancer	[111]

**Table 2:** Drugs that regulate ERK5 in bone diseases.

Drug	Mechanism	Disease	Reference
Nitrogen-containing bisphosphonates	Induce ERK5 phosphorylation and ERK5-dependent gene expression	Osteoporosis	[172]
mangiferin	Upregulated AXL	Osteoporosis	[202]
Vildagliptin	Upregulated AXL	Osteoporosis	[203]
TG02	block CDKs 1, 2, and 9 as well as ERK5 activity	Multiple myeloma	[198]
